# Awareness of Multisystem Inflammatory Syndrome in Children Among US Parents: A Cross-Sectional Survey

**DOI:** 10.1093/ofid/ofad476

**Published:** 2023-09-21

**Authors:** Lyndsey D Cole, E Adrianne Hammershaimb, Yuanyuan Liang, Megan A Hendrich, Dhiman Das, Robert Petrin, James D Campbell, Sean O’Leary, Jessica R Cataldi

**Affiliations:** Section of Infectious Diseases, Department of Pediatrics, University of Colorado School of Medicine, Aurora, Colorado, USA; Section of Rheumatology, Department of Pediatrics, University of Colorado School of Medicine, Aurora, Colorado, USA; Center for Vaccine Development and Global Health, University of Maryland School of Medicine, Baltimore, Maryland, USA; Division of Infectious Diseases and Tropical Pediatrics, Department of Pediatrics, University of Maryland School of Medicine, Baltimore, Maryland, USA; Center for Vaccine Development and Global Health, University of Maryland School of Medicine, Baltimore, Maryland, USA; Department of Epidemiology and Public Health, University of Maryland School of Medicine, Baltimore, Maryland, USA; Ipsos US Public Affairs, Washington, District of Columbia, USA; Ipsos US Public Affairs, Washington, District of Columbia, USA; Ipsos US Public Affairs, Washington, District of Columbia, USA; Center for Vaccine Development and Global Health, University of Maryland School of Medicine, Baltimore, Maryland, USA; Division of Infectious Diseases and Tropical Pediatrics, Department of Pediatrics, University of Maryland School of Medicine, Baltimore, Maryland, USA; Section of Infectious Diseases, Department of Pediatrics, University of Colorado School of Medicine, Aurora, Colorado, USA; Adult and Child Center for Health Outcomes Research and Delivery Science, University of Colorado School of Medicine, Aurora, Colorado, USA; Section of Infectious Diseases, Department of Pediatrics, University of Colorado School of Medicine, Aurora, Colorado, USA; Adult and Child Center for Health Outcomes Research and Delivery Science, University of Colorado School of Medicine, Aurora, Colorado, USA

**Keywords:** COVID-19, MIS-C, pediatrics, survey

## Abstract

**Background:**

Little is known about parental awareness of multisystem inflammatory syndrome in children (MIS-C), a rare but severe sequela of severe acute respiratory syndrome coronavirus 2 (SARS-CoV-2) infection.

**Methods:**

Via a nationally representative, cross-sectional survey of US parents conducted via Ipsos KnowledgePanel from October to November 2021, we used bivariate and multivariable analyses to describe and identify demographic variables associated with parental knowledge of and attitudes toward MIS-C and to examine associations with perceived coronavirus disease 2019 (COVID-19) severity and susceptibility.

**Results:**

Response rate was 64.2% (3230/5034). Thirty-two percent of respondents had heard of MIS-C. After adjustment, higher educational level (compared to high school degree; some college: odds ratio [OR], 2.00 [95% confidence interval {CI}, 1.44–2.77]; bachelor's degree or higher: OR, 3.14 [95% CI, 2.26–4.35]), being a healthcare worker (OR, 1.82 [95% CI, 1.37–2.42]), having a child with a chronic medical condition (OR, 1.62 [95% CI, 1.22–2.14]), and experience with more severe COVID-19 (OR, 1.46 [95% CI, 1.14–1.86]) were associated with MIS-C awareness. Respondents with a child aged 12–17 years were less likely to be aware of MIS-C compared to those without (OR, 0.78 [95% CI, .63–.96]), as were male respondents (OR, 0.56 [95% CI, .46–.69]) and respondents aged 18–34 years (OR, 0.72 [95% CI, .54–.94]) compared to those aged 35–44 years. Awareness of MIS-C was associated with higher perceived COVID-19 severity and susceptibility (regression coefficients, 0.18 [95% CI, .10–.25], *P* < .001; 0.19 [95% CI, .11–.28], *P* < .001, respectively).

**Conclusions:**

This survey highlights the need to increase parental awareness of MIS-C. Future studies should explore how education regarding MIS-C as a complication of SARS-CoV-2 infection could improve understanding of pediatric disease severity and susceptibility.

## Teaser

Through a nationally representative survey, we found that only one-third of US parents were aware of multisystem inflammatory syndrome in children (MIS-C). MIS-C awareness varied across demographics and was associated with higher perceived COVID-19 severity and susceptibility.

Multisystem inflammatory syndrome in children (MIS-C), first described in April 2020, is a pediatric hyperinflammatory illness associated with preceding severe acute respiratory syndrome coronavirus 2 (SARS-CoV-2) infection or exposure [1, 2]. As of 31 May 2023, the Centers for Disease Control and Prevention (CDC) has reported 9480 MIS-C cases and 79 deaths in the United States (US), with cases peaking in January 2021 [3]. In April–June 2020, estimated MIS-C incidence across 7 US jurisdictions was 316 per 1 000 000 SARS-CoV-2 infections in people <21 years of age [4]. The true incidence of MIS-C may be higher because healthcare workers may have had less familiarity with diagnosing MIS-C during those early months of the pandemic. Regardless, MIS-C poses a small but serious risk to children and is one of many reasons supporting pediatric coronavirus disease 2019 (COVID-19) immunization to prevent SARS-CoV-2 infection and associated complications [5, 6].

MIS-C is an exception to the generally lower morbidity and mortality from SARS-CoV-2–related illnesses in children compared to adults [7]. Patients with MIS-C are typically previously healthy children who present with high fevers and multiorgan involvement, including gastrointestinal symptoms, mucocutaneous findings, respiratory distress, and hemodynamic instability with cardiac dysfunction [8, 9]. Patients frequently require intensive care, vasoactive medications, and respiratory support. Complications can include persistent cardiac abnormalities and, rarely, death [8–12]. Treatment includes immunomodulating medications to abort the hyperinflammatory process [13]. The median age for MIS-C is 9 years, with nearly half of all cases occurring between age 5 and 11 years [3]. Racial and ethnic disparities in MIS-C incidence have been observed, with more than half of all reported cases occurring in patients who are Black/non-Hispanic or Hispanic/Latino [3, 14–16].

To date, no study has evaluated parental awareness of MIS-C. Understanding parental knowledge that this disease exists and is a complication of COVID-19 is important because MIS-C can mimic other conditions, progresses quickly, and requires urgent medical care. Additionally, knowledge of this life-threatening complication of COVID-19 in children may increase parents’ willingness to have their children vaccinated against COVID-19. Our objectives in this study were to describe parental knowledge of and attitudes toward MIS-C, identify demographic variables associated with knowledge of MIS-C, and assess whether knowledge of MIS-C was associated with differences in parents’ perceived disease severity and susceptibility for COVID-19 in children.

## METHODS

From 26 October 26 through 30 November 2021, a cross-sectional online survey of a nationally representative sample of US parents of children <18 years old was conducted via the Ipsos KnowledgePanel. For purposes of this study, “parent” refers to a parent or guardian who makes medical decisions for a child <18 years old in their household. The affiliated institutional review boards, including the CDC, determined the study as non–human subject research. The survey instrument (Supplementary File 1), developed in collaboration with CDC, collected demographic information and assessed parental knowledge and opinions regarding COVID-19. Respondents were asked if they had heard of MIS-C. If they had, they were presented with 2 statements assessing attitudes toward MIS-C (“It would be bad if my child got MIS-C” and “I am worried about the possibility of my child getting MIS-C”) with response options on a 4-point unipolar Likert-type scale (do not agree, somewhat agree, strongly agree, or very strongly agree). To assess personal experiences with COVID-19, respondents were asked whether they themselves or any adult or child they knew had had COVID-19. Respondents were then asked to indicate the highest level illness of severity among those COVID-19 cases. Response options for COVID-19 severity included no symptoms, mild symptoms, moderate symptoms, needing to be hospitalized, needing critical or intensive care, and, for experiences with COVID-19 in others, dying from their illness. The survey also included items designed around the Health Belief Model domains of disease severity and disease susceptibility related to COVID-19 [17]. The finalized English questionnaire was translated by 2 native speakers using a 2-person process that involved initial translation to Spanish by 1 individual and review by the second person. After the translated questionnaire was formatted, the translation team reviewed the Spanish content, grammar, and survey display against the English questionnaire and provided corrections. The formatted Spanish survey was then reviewed by 2 additional native Spanish speakers before survey distribution.

Ipsos KnowledgePanel is a probability-based web panel designed to provide a nationally representative sample of the US population. It uses an address-based sampling recruitment methodology based on the US Postal Service's Delivery Sequence File [18]. Stratified random sampling is used to match the geodemographic composition of the US adult population. To recruit Spanish-language-dominant panelists, supplemental dual-frame random-digit-dialing sampling is used. All panel members receive privacy and confidentiality protections, and if needed, a web-enabled device and free Internet service. Eligible panel members for the current study were English- or Spanish-speaking US adult healthcare decision-makers for at least 1 child <18 years of age in their household. Survey participants received an email invitation, reminder emails, and a standard incentive of points (redeemable for cash or other prizes) or sweepstakes entries. An extra $5–$10 incentive was offered to hard-to-reach respondents.

We excluded from the sample respondents who completed the survey in <25% of the median survey completion time and who skipped ≥50% of eligible questions, as well as those whose reported age and sex did not match their panel profile demographics. Weights allowing the projection of all results to the general population of US parents were computed [18]. Details regarding weighting methods and data cleaning can be found in a previous analysis of related survey data [19]. All analyses presented in this article reflect the application of survey weights.

Responses regarding personal experiences of COVID-19 in the respondent, adults the respondent knew, and children the respondent knew were combined to create a single dichotomous variable based on severity of their experiences with COVID-19 (either “no experience, no symptoms, or mild symptoms” or “moderate symptoms, hospitalization/critical care, or death”). For survey items designed to reflect Health Belief Model domains of disease severity and susceptibility, we used weighted Cronbach α to evaluate for internal domain consistency. If Cronbach α was ≥0.7, we calculated an average composite score for each domain for use in subsequent analyses.

Among all respondents, bivariate associations were measured between awareness of MIS-C and each predictor of interest, including demographic factors and personal experiences with COVID-19, using odds ratios (ORs) and corresponding 95% confidence intervals (CIs). Multivariable logistic regression models were then constructed with the primary outcome of awareness of MIS-C. Significant factors (*P* < .05) from the bivariate analyses were included in multivariable models. Following the fitting of a model to evaluate net correlates of MIS-C awareness for the overall sample, separate models for MIS-C awareness were generated for parents of children aged 0–4 years, parents of children aged 5–11 years, and parents of children aged 12–17 years. For the 60% of respondents with multiple children, they may appear in 2 or more subgroup models based on their children's ages. These age groups roughly align with pediatric COVID-19 vaccine development groups and were selected to reflect preschool children, school-aged children, and adolescents, who have unique epidemiology of COVID-19 disease and MIS-C.

To assess how awareness of MIS-C relates to perceived COVID-19 disease severity and susceptibility in children, unadjusted linear regressions were performed first. These associations were measured separately for all respondents, those with children aged 0–4 years, those with children aged 5–11 years, and those with children aged 12–17 years. Two separate multivariable linear regression models were then generated for the 2 outcomes, perceived COVID-19 severity and perceived COVID-19 susceptibility. Each of these multivariable models included awareness of MIS-C, demographic factors, and personal experiences with COVID-19 as potential predictors of perceived COVID-19 severity and perceived COVID-19 susceptibility. All analyses were conducted in R version 4.1.2 software for Windows using package “survey.”

## RESULTS

The survey response rate was 64.2% (3230/5034). Of these 3230 respondents, 3082 respondents qualified for the survey as parental healthcare decision-makers for children <18 years of age, and 3042 respondents qualified for analysis after data cleaning. Sampling weights were generated for this sample of 3042 respondents. Weighted and unweighted survey respondent characteristics are presented in a previous analysis of related data from the same survey sample [19]. The weighted percentage of respondents who said they had heard of MIS-C was 32%. Among parents aware of MIS-C, 97% somewhat, strongly, or very strongly agreed “it would be bad if [their] child got MIS-C,” and 81% agreed they were “worried about the possibility of [their] child getting MIS-C.”

Weighted bivariate analyses indicated that among all respondents, those who were aware of MIS-C were more likely to be female, aged 35–44 years (compared to aged 18–34 years), White/non-Hispanic (compared to Hispanic and Black/non-Hispanic), English-speaking, and healthcare workers (Table 1). Respondents aware of MIS-C were also more likely to have a higher income, higher education, a child with a chronic medical condition, and personal experience with more severe COVID-19 in themselves or someone they knew. Respondents with a child aged 12–17 years were less likely to be aware of MIS-C compared to respondents without a child aged 12–17 years.

**Table 1.** Bivariate Analysis of Characteristics Associated With Awareness of Multisystem Inflammatory Syndrome in Children Among All Respondents

Characteristic	Aware of MIS-C, Weighted %	Odds Ratio (95% CI)	*P* Value
Age of parent, y			
35–44	34	1	
18–34	26	0.69 (.54–.87)	.002
45–54	34	0.97 (.80–1.19)	.80
≥55	30	0.82 (.58–1.17)	.28
Gender			
Female	35	1	
Male	27	0.69 (.57–.82)	<.001
Age of children			
0–4 y			
No	31	1	
Yes	33	1.13 (.94–1.37)	.12
5–11 y			
No	31	1	
Yes	32	1.03 (.86–1.22)	.77
12–17 y			
No	33	1	
Yes	30	0.84 (.71–1.00)	.05
Race/ethnicity			
White, non-Hispanic	35	1	
Black, non-Hispanic	25	0.60 (.42–.87)	.007
Other, non-Hispanic	35	1.00 (.70–1.43)	1
Hispanic	22	0.53 (.41–.68)	<.001
≥2 races, non-Hispanic	46	1.59 (1.00–2.53)	.05
Survey language			
English	33	1	
Spanish	14	0.34 (.22–.52)	<.001
Education			
High school	18	1	
Less than high school	15	0.83 (.52–1.33)	.44
Some college	32	2.19 (1.61–2.96)	<.001
Bachelor's degree or higher	44	3.51 (2.67–4.62)	<.001
Healthcare worker			
No	30	1	
Yes	50	2.36 (1.79–3.11)	<.001
Household income			
$25 000-$74 999	24	1	
<$25 000	18	0.70 (.49–1.02)	.06
≥$75 000	37	1.85 (1.50–2.27)	<.001
Census region			
Northeast	32	1	
Midwest	30	0.91 (.69–1.20)	.52
South	31	0.95 (.73–1.23)	.68
West	33	1.01 (.77–1.33)	.93
Urbanicity			
Suburban	31	1	
Urban	33	1.09 (.90–1.33)	.37
Rural	32	1.05 (.82–1.34)	.69
Child with chronic medical condition			
No	30	1	
Yes	42	1.70 (1.30–2.21)	<.001
Personal COVID-19 experience^a^			
None/no or mild symptoms	23	1	
Moderate symptoms, hospitalization, death	34	1.73 (1.37–2.17)	<.001

Abbreviations: CI, confidence interval; COVID-19, coronavirus disease 2019; MIS-C, multisystem inflammatory syndrome in children.

a Most severe level of illness due to COVID-19 in the respondent, adults they knew, and/or children they knew.

**Figure 1.**
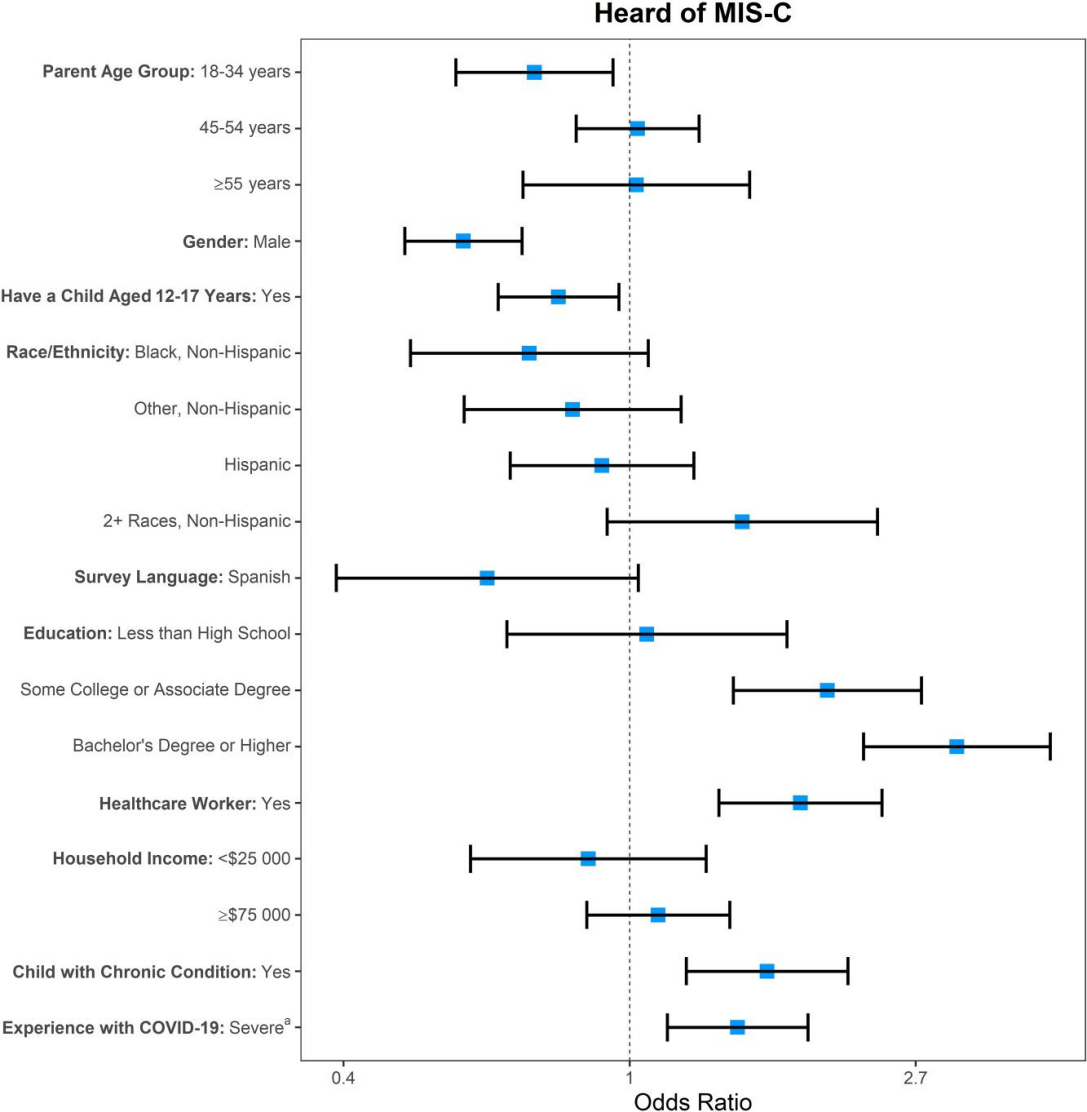
Weighted multivariable analysis of characteristics associated with awareness of multisystem inflammatory syndrome in children (MIS-C) among all respondents. Reference categories: Age (years) = 35–44; Gender = Female; Child aged 12–17 y = No; Race/ethnicity = White, non-Hispanic; Survey language = English; Education = High school degree; Healthcare worker = No; Household income = $25 000–$74 999; Child with chronic medical condition = No; Personal coronavirus disease 2019 (COVID-19) experience = None/no or mild symptoms only. ^a^Personal COVID-19 experience includes the most severe level of illness due to COVID-19 in the respondent, adults they knew, and/or children they knew. “Severe” refers to moderate symptoms, hospitalization, or death from COVID-19.

From the total sample weighted multivariable logistic regression analysis, having a higher educational level (some college or associate degree: OR, 2.00 [95% CI, 1.44–2.77]; bachelor's degree or higher: OR, 3.14 [95% CI, 2.26–4.35] compared to high school degree), being a healthcare worker (OR, 1.82 [95% CI, 1.37–2.42]), having a child with a chronic medical condition (OR, 1.62 [95% CI, 1.22–2.14]), and having personal experience with more severe COVID-19 (OR, 1.46 [95% CI, 1.14–1.86]) were associated with MIS-C awareness (Figure 1; Supplementary Table 1). Respondents with a child aged 12–17 years were less likely to be aware of MIS-C (OR, 0.78 [95% CI, .63–.96]) as were male respondents (OR, 0.56 [95% CI, .46–.69]) and respondents aged 18–34 years (OR, 0.72 [95% CI, .54–.94]) compared to those aged 35–44 years.

The sample was broken out by age subgroups and used in separate multivariate regression analyses, which demonstrated that parents of children across all age groups were more likely to be aware of MIS-C if they had higher levels of education or were healthcare workers (Supplementary Table 1). Parents of children aged 0–4 years were less likely to be aware of MIS-C if they were 18–34 years of age. Respondents with children aged 5–11 or 12–17 years were also more likely to be aware of MIS-C if they were female, had a child with a chronic medical condition, or had personal experience with more severe COVID-19. Parents with children aged 12–17 years were less likely to be aware of MIS-C if they were Black/non-Hispanic or other/non-Hispanic compared to White/non-Hispanic respondents.

**Figure 2.**
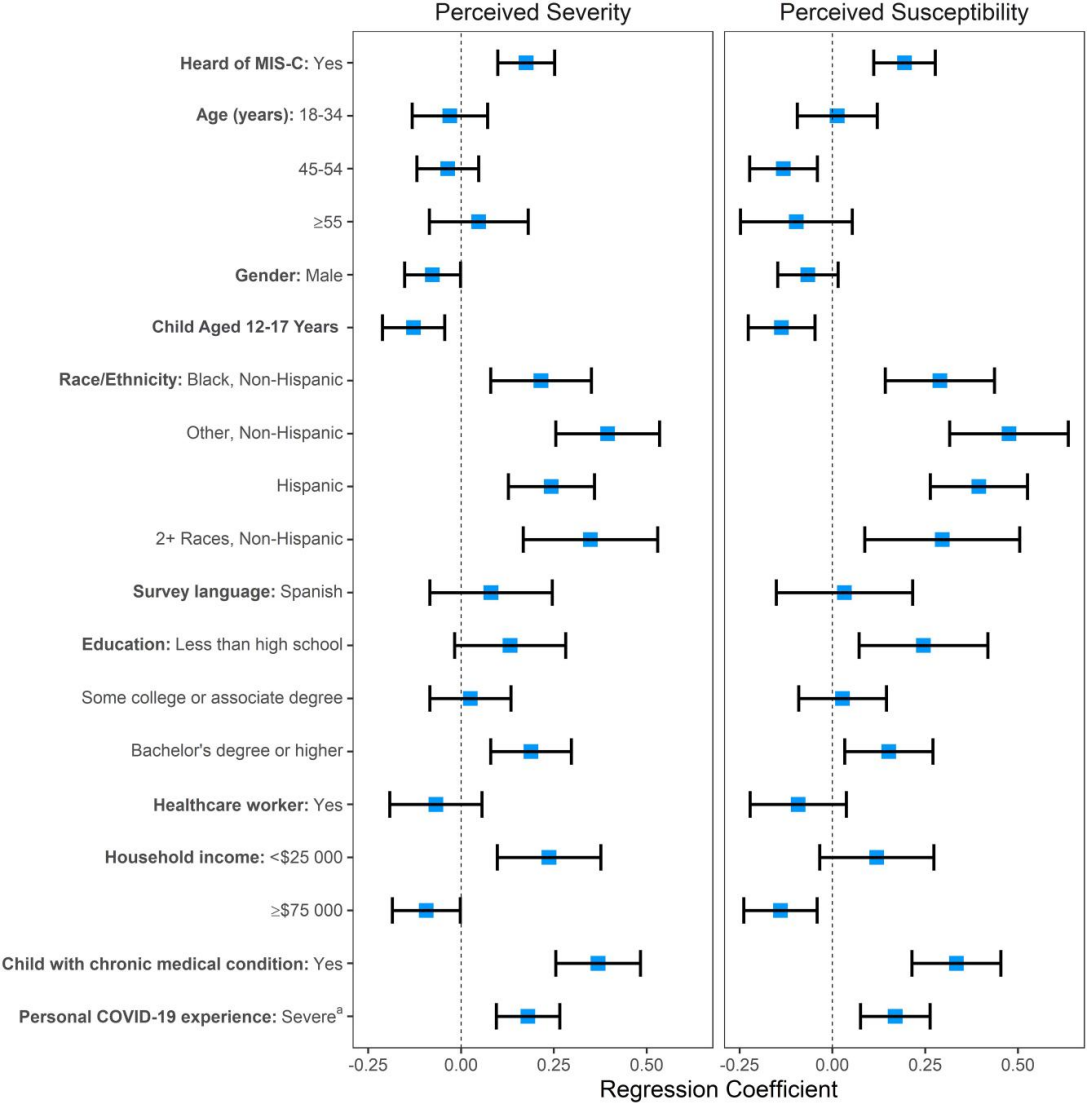
Weighted multivariable analysis of characteristics associated with perceived coronavirus disease 2019 (COVID-19) severity and susceptibility including awareness of multisystem inflammatory syndrome in children (MIS-C). Reference categories: Heard of MIS-C = No; Age (years) = 35–44; Gender = Female; Child aged 12–17 y = No; Race/ethnicity = White, non-Hispanic; Survey language = English; Education = High school degree; Healthcare worker = No; Household income = $25 000–$74 999; Child with chronic medical condition = No; Personal COVID-19 experience = None/no or mild symptoms only. ^a^Personal COVID-19 experience includes the most severe level of illness due to COVID-19 in the respondent, adults they knew, and/or children they knew. “Severe” refers to moderate symptoms, hospitalization, or death from COVID-19.

In a series of ordinary least squares regression models that regressed perceived COVID-19 severity on MIS-C awareness overall and for parents of children in each age group separately, awareness of MIS-C was significantly associated with higher perceived COVID-19 severity among all respondents (regression coefficient, 0.20 [95% CI, .12–.27], *P* < .001, indicating that the mean composite perceived disease severity score [range, 1–4] was 0.2 units higher among those aware of MIS-C compared to those not aware of MIS-C) (Table 2). This association was similar in the subgroup analyses for parents of children aged 0–4 years, 5–11 years, and 12–17 years. In a companion series of ordinary least squares regression models that regressed perceived COVID-19 susceptibility on awareness of MIS-C, awareness of MIS-C was also significantly associated with higher perceived COVID-19 susceptibility among all respondents (regression coefficient, 0.17 [95% CI, .09–.25], *P* < .001) and for parents of children aged 0–4 and 5–11 years, but not for parents of children aged 12–15 years (Table 2). After adjustment for demographic variables, among all respondents, awareness of MIS-C remained significantly associated with both perceived COVID-19 severity and susceptibility, as did several demographic factors and personal experience with more severe COVID-19 (Figure 2; Supplementary Table 2).

**Table 2.** Unadjusted Effect of Awareness of Multisystem Inflammatory Syndrome in Children on Perceived Coronavirus Disease 2019 Susceptibility and Severity Among All Respondents and by Age Group of Children

Aware of MIS-C	All Respondents		Children Aged 0–4 y		Children Aged 5–11 y		Children Aged 12–17 y	
	Coeff^a^ (95% CI)	*P* Value	Coeff (95% CI)	*P* Value	Coeff (95% CI)	*P* Value	Coeff (95% CI)	*P* Value
Perceived COVID-19 severity								
Yes vs no	0.20 (.12–.27)	<.001	0.16 (.03–.30)	.02	0.20 (.10–.30)	<.001	0.15 (.05–.25)	.003
Perceived COVID-19 susceptibility								
Yes vs no	0.17 (.09–.25)	<.001	0.21 (.07–.35)	.004	0.19 (.08–.29)	.001	0.06 (−.05 to .17)	.26

Abbreviations: CI, confidence interval; coeff, weighted regression coefficient; COVID-19, coronavirus disease 2019; MIS-C, multisystem inflammatory syndrome in children.

a Weighted regression coefficient represents the difference in the mean composite COVID-19 severity (or COVID-19 susceptibility) score (range, 1–4). For example, a coefficient of 0.2 indicates that the mean perceived COVID-19 severity score was 0.2 units higher among respondents aware of MIS-C compared with those not aware of MIS-C.

## DISCUSSION

MIS-C is an important cause of morbidity and mortality in children associated with SARS-CoV-2 infection. One and a half years after initial descriptions of MIS-C, this October–November 2021 survey demonstrated that only one-third of parents were aware of its existence.

Among respondents who had children 12–17 years of age, those with Black/non-Hispanic and Other/non-Hispanic race/ethnicity were less likely to be aware of MIS-C in multivariable analysis. Overall awareness of MIS-C was low for Black and Hispanic respondents (22%–25%). Additional racial/ethnic and language-related differences in MIS-C awareness were identified in bivariate but not multivariable analysis. Throughout most of the pandemic, incidence of SARS-CoV-2 infection and incidence of MIS-C have been higher among children who are Hispanic/Latinx and Black/African American [3, 14–16, 20, 21]. While it has been considered whether differences in MIS-C incidence could be due solely to racial/ethnic disparities in SARS-CoV-2 infection, analysis of data from 369 US counties from March 2020 to February 2021 found that rates of MIS-C were 207% of the expected rate (based on incidence of SARS-CoV-2 infection) in Black/non-Hispanic children, 68% of the expected rate in White/non-Hispanic children, and 102% of the expected rate among Hispanic children [14]. Considering racial and ethnic disparities in risk of SARS-CoV-2 infection and MIS-C, efforts to increase awareness of MIS-C must include and prioritize parents of Black and Hispanic children as well as Spanish-speaking parents.

In addition to parent race and ethnicity, we assessed how the ages of the respondents’ children affected their awareness of MIS-C. About 50% of cases of MIS-C occur in children aged 5–11, but respondents with children in this age group were no more likely to have heard of MIS-C and overall awareness was low at 32% [3]. Respondents with children aged 12–17 years were less likely to have heard of MIS-C; however, reasons for this are unclear. Awareness of MIS-C was associated with increased perceived COVID-19 susceptibility in respondents with younger children, but not in respondents with children aged 12–17 years, and being a parent of a child aged 12–17 years was associated with lower perceived COVID-19 severity and susceptibility. Although MIS-C is less common among adolescents, it can still occur. It is important to improve awareness of MIS-C among parents of older children to ensure they understand the range of negative consequences associated with SARS-CoV-2 infection.

Other demographic factors associated with lower awareness of MIS-C included being a male parent, being 18–34 years of age, and having a high school education (compared to some college or associate degree and bachelor's degree or higher). Male respondents also reported lower perceived COVID-19 severity in children. Communication efforts through public health messaging and conversations with healthcare providers should include parents of all genders, noting that how a male parent perceives MIS-C and COVID-19 risks may influence healthcare decisions for his child. Messages about MIS-C should also be tailored to reach younger parents and parents with less education. Younger parents may be more likely to use online resources to obtain information about vaccine-preventable diseases, and a multimedia approach will likely reach more people, though healthcare providers remain essential messengers [22]. Materials and messages about MIS-C should be designed with health literacy in mind and must be accessible to parents across educational backgrounds.

With the development of effective COVID-19 vaccines, including for children, MIS-C represents a complication of a now vaccine-preventable illness [23, 24]. Understanding and awareness of potential complications of COVID-19 is important for parents making decisions about whether to vaccinate their children against COVID-19. Focusing on disease risk has been shown to be an effective strategy in increasing vaccine uptake in the pre-COVID-19 era [25].

In previous analysis of our related survey data also collected from October–November 2021, we assessed the association of awareness of MIS-C and parental intent to vaccinate children against COVID-19 [19]. Respondents aware of MIS-C were more likely to report they were somewhat/very likely to accept COVID-19 vaccination for their child although this association was no longer significant in analysis adjusting for demographic and other factors [19]. On multivariable analysis, higher perceived COVID-19 disease severity in children was associated with increased vaccination intent among parents of children aged 0–4 and 5–11 years [19]. Here, we have shown that awareness of MIS-C influences perception of disease severity. It is important to educate parents about risks associated with COVID-19 including MIS-C, and by association informing parents that benefits associated with vaccination include decreased risk of COVID-19 and MIS-C [23, 24, 26, 27].

Limitations of this study include the cross-sectional design with a single time point in October–November 2021, given that MIS-C awareness and attitudes may change over time as the pandemic continues and experience with COVID-19 evolves. Perceptions of COVID-19 severity and susceptibility in children and personal experiences with COVID-19 may also change over time. Collapsing response categories to reflect personal experience with COVID-19 as a dichotomous variable was pursued to simplify interpretation of the analysis but may have obscured more nuanced effects of personal COVID-19 experience across response categories. In addition, respondents’ interpretation of mild and moderate symptoms may have varied. Because MIS-C has been described using a variety of different terms in both English and Spanish, some respondents may have indicated a lack of knowledge about MIS-C when they were aware of MIS-C by another name. A large proportion of respondents had children in multiple age categories, potentially making comparisons of responses between parents of children in different age groups somewhat more challenging but also reflective of real-life families. We did not conduct any analyses related to number of children or household size. Survey responses may have been subject to social desirability bias. We relied on self-report and did not independently assess actual behaviors or experiences. Additionally, at the onset of this survey administration, the Pfizer-BioNTech COVID-19 vaccine was authorized for emergency use in the US for ages 12 years and older; 3 days into survey administration, this was expanded to include ages 5 years and older [24]. Results from this study are not generalizable to parents who speak neither English nor Spanish or to non-US populations.

In conclusion, this survey highlights the need for further parental awareness of MIS-C, particularly among communities at highest risk. We found that parents who were aware of MIS-C had significantly higher perception of COVID-19 disease severity and susceptibility in children. Future studies are necessary to explore how education regarding MIS-C as a complication of SARS-CoV-2 infection could help improve parental understanding of disease severity and susceptibility and how this may impact decision-making regarding COVID-19 vaccination for children. This work should be equitable and inclusive of the most vulnerable populations of children who are at higher risk of SARS-CoV-2 infection and MIS-C. While MIS-C has been reported less frequently in the US in 2023 [3], this study provides valuable understanding of this severe complication of COVID-19 from a timepoint when MIS-C was more common. This information could inform future messaging for time periods when SARS-CoV-2 and MIS-C activity may increase. Our findings also highlight the need to improve parental awareness of rare but severe complications of vaccine-preventable diseases now and in the future.

## Supplementary Material

ofad476_Supplementary_Data

## Supplementary Data

Supplementary materials are available at *Open Forum Infectious Diseases* online. Consisting of data provided by the authors to benefit the reader, the posted materials are not copyedited and are the sole responsibility of the authors, so questions or comments should be addressed to the corresponding author.

## Notes

***Disclaimer.*** The Centers for Disease Control and Prevention (CDC) contributed to the early-stage design of the study but had no role in the conduct of the study, review of the data, or manuscript preparation. The findings and conclusions in this report are those of the authors and do not represent the official position of the CDC.

***Financial support.*** This work was supported by the US Centers for Disease Control and Prevention.
