# Supplementary material for: Awareness of Multisystem Inflammatory Syndrome in Children Among US Parents: A Cross-Sectional Survey
Source: Open Forum Infect Dis. 2023 Sep 21;10(10):ofad476. doi: 10.1093/ofid/ofad476 (PMC10546954; doi:10.1093/ofid/ofad476)
Supplement: ofad476_Supplementary_Data [file ofad476_supplementary_data.zip › MIS-C Survey Supplemental Table 1 06.21.23.docx]

| **Respondent Characteristic** | | **All Respondents** | | **Children Aged 0-4** | | **Children Aged 5-11** | | **Children Aged 12-17** | |
| --- | --- | --- | --- | --- | --- | --- | --- | --- | --- |
|  |  | **Odds Ratio (95% CI)** | ***P* value** | **Odds Ratio (95% CI)** | ***P* value** | **Odds Ratio (95% CI)** | ***P* value** | **Odds Ratio (95% CI)** | ***P* value** |
| Age (years) | 35-44 | 1 |  | 1 |  | 1 |  | 1 |  |
|  | 18-34 | 0.72 (0.54, 0.94) | 0.02 | 0.68 (0.49, 0.96) | 0.03 | 0.76 (0.53, 1.09) | 0.14 | 0.69 (0.36, 1.33) | 0.27 |
|  | 45-54 | 1.03 (0.83, 1.27) | 0.80 | 1.10 (0.57, 2.12) | 0.78 | 1.11 (0.82, 1.49) | 0.50 | 1.08 (0.82, 1.41) | 0.59 |
|  | 55+ | 1.02 (0.69, 1.52) | 0.91 | 3.91 (0.52, 29.2) | 0.18 | 0.85 (0.30, 2.42) | 0.77 | 1.13 (0.72, 1.76) | 0.59 |
| Gender | Female | 1 |  | 1 |  | 1 |  | 1 |  |
|  | Male | 0.56 (0.46, 0.69) | <0.001 | 0.76 (0.52, 1.10) | 0.14 | 0.49 (0.37, 0.66) | <0.001 | 0.46 (0.35, 0.61) | <0.001 |
| Child aged 12-17 years | No | 1 |  |  |  |  |  |  |  |
|  | Yes | 0.78 (0.63, 0.96) | 0.02 |  |  |  |  |  |  |
| Race/ Ethnicity | White, Non-Hispanic | 1 |  | 1 |  | 1 |  | 1 |  |
|  | Black, Non-Hispanic | 0.70 (0.46, 1.07) | 0.10 | 0.64 (0.30, 1.37) | 0.25 | 0.65 (0.35, 1.22) | 0.18 | 0.57 (0.33, 0.97) | 0.04 |
|  | Other, Non-Hispanic | 0.82 (0.56, 1.20) | 0.30 | 1.26 (0.66, 2.37) | 0.48 | 0.63 (0.35, 1.11) | 0.11 | 0.57 (0.32, 0.98) | 0.04 |
|  | Hispanic | 0.91 (0.66, 1.25) | 0.55 | 0.69 (0.38, 1.27) | 0.23 | 0.88 (0.57, 1.37) | 0.58 | 0.95 (0.62, 1.44) | 0.80 |
|  | 2+ Races, Non-Hispanic | 1.48 (0.92, 2.38) | 0.10 | 1.20 (0.58, 2.48) | 0.62 | 1.04 (0.51, 2.12) | 0.90 | 1.36 (0.69, 2.65) | 0.37 |
| Survey language | English | 1 |  | 1 |  | 1 |  | 1 |  |
|  | Spanish | 0.61 (0.36, 1.03) | 0.06 | 0.49 (0.15, 1.62) | 0.24 | 0.65 (0.32, 1.33) | 0.24 | 0.56 (0.30, 1.07) | 0.08 |
| Education | High school | 1 |  | 1 |  | 1 |  | 1 |  |
|  | Less than high school | 1.06 (0.65, 1.73) | 0.81 | 0.63 (0.23, 1.73) | 0.37 | 0.86 (0.43, 1.73) | 0.68 | 1.38 (0.76, 2.48) | 0.29 |
|  | Some college or associate degree | 2.00 (1.44, 2.77) | <0.001 | 1.79 (0.97, 3.31) | 0.06 | 1.50 (0.94, 2.38) | 0.09 | 2.03 (1.36, 3.03) | 0.001 |
|  | Bachelor’s degree or higher | 3.14 (2.26, 4.35) | <0.001 | 1.98 (1.06, 3.69) | 0.03 | 3.03 (1.88, 4.89) | <0.001 | 3.30 (2.24, 4.85) | <0.001 |
| Healthcare worker | No | 1 |  | 1 |  | 1 |  | 1 |  |
|  | Yes | 1.82 (1.37, 2.42) | <0.001 | 2.04 (1.25, 3.33) | 0.004 | 1.88 (1.25, 2.83) | .002 | 1.60 (1.09, 2.35) | 0.02 |
| Household income | $25,000-$74,999 | 1 |  | 1 |  | 1 |  | 1 |  |
|  | < $25,000 | 0.87 (0.57, 1.31) | 0.49 | 0.96 (0.46, 1.98) | 0.91 | 0.78 (0.43, 1.40) | 0.40 | 0.81 (0.47, 1.40) | 0.46 |
|  | ≥ $75,000 | 1.10 (0.86, 1.42) | 0.43 | 1.41 (0.89, 2.25) | 0.15 | 1.24 (0.87, 1.77) | 0.22 | 1.03 (0.74, 1.42) | 0.88 |
| Child with chronic medical condition | No | 1 |  | 1 |  | 1 |  | 1 |  |
|  | Yes | 1.62 (1.22, 2.14) | 0.001 | 1.53 (0.87, 2.67) | 0.14 | 1.72 (1.19, 2.48) | 0.004 | 1.74 (1.21, 2.51) | 0.003 |
| Personal COVID-19 experience^a^ | None/no or mild symptoms only | 1 |  | 1 |  | 1 |  | 1 |  |
|  | Moderate symptoms, hospitalization, or death | 1.46 (1.14, 1.86) | 0.003 | 1.41 (0.92, 2.16) | 0.11 | 1.45 (1.03, 2.06) | 0.04 | 1.42 (1.02, 1.97) | 0.04 |

**Supplemental Table 1:** Weighted Multivariable Analysis of Characteristics Associated with Awareness of MIS-C Among All Respondents and By Age Group of Children**.**

^a^ Most severe level of illness due to COVID-19 in the respondent, adults they knew, and/or children they knew.

CI=confidence interval.
