# Supplementary material for: Awareness of Multisystem Inflammatory Syndrome in Children Among US Parents: A Cross-Sectional Survey
Source: Open Forum Infect Dis. 2023 Sep 21;10(10):ofad476. doi: 10.1093/ofid/ofad476 (PMC10546954; doi:10.1093/ofid/ofad476)
Supplement: ofad476_Supplementary_Data [file ofad476_supplementary_data.zip › MIS-C Survey Supplemental Table 2 06.21.23.docx]

| **Respondent Characteristic** | | **Perceived COVID-19 Severity** | | **Perceived COVID-19 Susceptibility** | |
| --- | --- | --- | --- | --- | --- |
|  |  | **Coeff (95% CI)** | ***P* value** | **Coeff (95% CI)** | ***P* value** |
| Awareness of MIS-C | No | 0 |  | 0 |  |
|  | Yes | 0.18 (0.10, 0.25) | <0.001 | 0.19 (0.11, 0.28) | <0.001 |
| Age (years) | 35-44 | 0 |  | 0 |  |
|  | 18-34 | -0.03 (-0.13, 0.07) | 0.56 | 0.01 (-0.09, 0.12) | 0.82 |
|  | 45-54 | -0.04 (-0.12, 0.05) | 0.40 | -0.13 (-0.22, -0.04) | 0.004 |
|  | 55+ | 0.05 (-0.09, 0.18) | 0.48 | -0.10 (-0.24, 0.05) | 0.21 |
| Gender | Female | 0 |  | 0 |  |
|  | Male | -0.08 (-0.15, 0.00) | 0.04 | -0.07 (-0.15, 0.02) | 0.11 |
| Child aged 12-17 years | No | 0 |  | 0 |  |
|  | Yes | -0.13 (-0.21, -0.04) | 0.003 | -0.14 (-0.23, -0.05) | 0.003 |
| Race/Ethnicity | White, Non-Hispanic | 0 |  | 0 |  |
|  | Black, Non-Hispanic | 0.22 (0.08, 0.35) | 0.002 | 0.29 (0.14, 0.44) | <0.001 |
|  | Other, Non-Hispanic | 0.39 (0.25, 0.53) | <0.001 | 0.48 (0.32, 0.64) | <0.001 |
|  | Hispanic | 0.24 (0.13, 0.36) | <0.001 | 0.39 (0.26, 0.53) | <0.001 |
|  | 2+ Races, Non-Hispanic | 0.35 (0.17, 0.53) | <0.001 | 0.30 (0.09, 0.50) | 0.005 |
| Survey language | English | 0 |  | 0 |  |
|  | Spanish | 0.08 (-0.08, 0.25) | 0.34 | 0.03 (-0.15, 0.22) | 0.73 |
| Education | High school | 0 |  | 0 |  |
|  | Less than high school | 0.13 (-0.02, 0.28) | 0.08 | 0.24 (0.07, 0.42) | 0.006 |
|  | Some college or associate degree | 0.03 (-0.08, 0.13) | 0.65 | 0.03 (-0.09, 0.14) | 0.65 |
|  | Bachelor’s degree or higher | 0.19 (0.08, 0.30) | 0.001 | 0.15 (0.03, 0.27) | 0.012 |
| Healthcare worker | No | 0 |  | 0 |  |
|  | Yes | -0.07 (-0.19, 0.06) | 0.28 | -0.09 (-0.22, 0.04) | 0.16 |
| Household income | $25,000-$74,999 | 0 |  | 0 |  |
|  | <$25,000 | 0.24 (0.10, 0.38) | 0.001 | 0.12 (-0.03, 0.27) | 0.13 |
|  | ≥$75,000 | -0.09 (-0.19, 0.00) | 0.04 | -0.14 (-0.24, -0.04) | 0.005 |
| Child with chronic medical condition | No | 0 |  | 0 |  |
|  | Yes | 0.37 (0.25, 0.48) | <0.001 | 0.33 (0.21, 0.45) | <0.001 |
| Personal COVID-19 experience^a^ | No experience/no or mild symptoms only | 0 |  | 0 |  |
|  | Moderate symptoms, hospitalization, or death | 0.18 (0.09, 0.27) | <0.001 | 0.17 (0.08, 0.26) | <0.001 |

**Supplemental Table 2:** Weighted Multivariable Analysis of Characteristics Associated with Perceived COVID-19 Severity and Susceptibility Including Awareness of MIS-C.

^a^ Most severe level of illness due to COVID-19 in the respondent, adults they knew, and/or children they knew.

CI=confidence interval; coeff=weighted regression coefficient.
