# Supplementary material for: Awareness of Multisystem Inflammatory Syndrome in Children Among US Parents: A Cross-Sectional Survey
Source: Open Forum Infect Dis. 2023 Sep 21;10(10):ofad476. doi: 10.1093/ofid/ofad476 (PMC10546954; doi:10.1093/ofid/ofad476)
Supplement: ofad476_Supplementary_Data [file ofad476_supplementary_data.zip › MIS-C Survey Supplemental File 1 Survey Instrument.docx]

**Supplemental File 1: Parental COVID-19 Vaccine Intention Survey Instrument**

1. In this survey, we would like to know what you, as a parent or guardian, think about routine childhood vaccines as well as COVID-19 and COVID-19 vaccines.
   1. First, are you a parent or guardian of a child aged 0 to 17 who lives in your household?
      - 1. Yes
        2. No
   2. How many children aged 0 to 17 in your household do you make healthcare decisions for?
      - 1. [TEXTBOX] children under 18 years old
   3. What is the age of each child who you make healthcare decisions for?
      - 1. Child 1: [TEXTBOX] years old
        2. Child 2: [TEXTBOX] years old
        3. (Continues for the number of children entered in the previous question)
   4. What is your relationship to the [child]/[children] who you make healthcare decisions for? Please select ALL that apply.
      - 1. Parent
        2. Grandparent
        3. Other family member
        4. Unrelated guardian
2. Next, we would like to know your opinions about routine childhood vaccines.
   1. How much do you agree or disagree with the following?
      1. *Statements:*
         1. All childhood vaccines offered by my [child’s]/[children’s] healthcare provider are beneficial.
         2. I do what my [child’s]/[children’s] healthcare provider recommends about vaccines.
         3. I am concerned about serious side effects of childhood vaccines.
         4. Getting vaccines is a good way to protect my [child]/[children] from disease.
         5. The information I receive about childhood vaccines from my [child’s]/[children’s] healthcare provider is reliable and trustworthy.
         6. Having my [child]/[children] vaccinated is important for the health of others in my community.
         7. Childhood vaccines are effective.
         8. Childhood vaccines are important for my [child’s]/[children’s] health.
         9. New vaccines carry more risks than older vaccines.
         10. I trust that FDA approval means that a vaccine is safe.
      2. *Responses:*
         1. Strongly agree
         2. Somewhat agree
         3. Somewhat disagree
         4. Strongly disagree
   2. During the flu season from July 2019 to May 2020 (before the COVID-19 pandemic started), did [your child]/[any of your children] get the flu vaccine?
      - 1. Yes
        2. No
        3. Not sure
        4. [My child was not]/[None of my children were] eligible to get a flu vaccine
3. The next section of this survey will focus on your experiences with and thoughts about COVID-19.
   1. How much do you rely on the following sources for information about COVID-19?
      1. *Statements:*
         1. TV
         2. Print or online news
         3. Radio or podcasts
         4. Social media
         5. Healthcare provider
         6. Government health website (such as CDC, FDA) or other reputable health website
         7. Friends and family
      2. *Responses:*
         1. Rely a lot
         2. Rely somewhat
         3. Do not rely on this
   2. Have you ever had COVID-19?
      - 1. Yes
        2. No
        3. Not sure
   3. [*If answer to Q4 was “Yes” or “Not sure” 🡪*] Which of the following best describes your symptoms when you [had]/[might have had] COVID-19?
      - 1. No symptoms
        2. Mild symptoms
        3. Moderate symptoms
        4. Severe symptoms
   4. [*If answer to Q4 was “Yes” or “Not sure” 🡪*] Did you need to be hospitalized?
      - 1. Yes
        2. No
   5. [*If answer to Q6 was “Yes” 🡪*] Did you require critical (ICU) care?
      - 1. Yes
        2. No
   6. Do you personally know anyone other than yourself who has had COVID-19?
      1. *Statements:*
         1. An adult who lives with me
         2. An adult who does **not** live with me
         3. [*If parent of child ages 12-17 🡪*] A child aged 12 to 17 who lives with me
         4. A child aged 12 to 17 who does **not** live with me
         5. [*If parent of child ages 5-11 🡪*] A child aged 5 to 11 who lives with me
         6. A child aged 5 to 11 who does **not** live with me
         7. [*If parent of child ages 0-4 🡪*] A child aged 0 to 4 who lives with me
         8. A child aged 0 to 4 who does **not** live with me
      2. *Responses:*
         1. Yes
         2. No
   7. [*If answer to Q8_1 or Q8_2 was “Yes” 🡪*] Thinking of all the adults you personally know who have had COVID-19, what was the most severe case that you’ve seen?
      - 1. No symptoms
        2. Mild symptoms
        3. Moderate symptoms
        4. Needing to be hospitalized
        5. Needing critical (ICU) care
        6. Dying from their illness
   8. [*If any answer to Q8_3 through Q8_8 was “Yes” 🡪*] Thinking of all the children you personally know who have had COVID-19, what was the most severe case that you’ve seen?
      - 1. No symptoms
        2. Mild symptoms
        3. Moderate symptoms
        4. Needing to be hospitalized
        5. Needing critical (ICU) care
        6. Dying from their illness
   9. Have you ever heard of MIS-C (multi-system inflammatory syndrome in children) related to COVID-19?
      - 1. Yes
        2. No
        3. Not sure
   10. [*If answer to Q11 was “Yes” 🡪*] [Has your child]/[Have any of your children] ever had MIS-C?
       - 1. Yes
         2. No
   11. [*If answer to Q11 was “Yes” 🡪*] How much do you agree with the following statements about **MIS-C** **in children**?
       1. *Statements:*
          1. I am worried about the possibility of my [child]/[children] getting MIS-C.
          2. It would be bad if my [child]/[children] got MIS-C.
       2. *Responses:*
          1. Very strongly agree
          2. Strongly agree
          3. Somewhat agree
          4. Do not agree
   12. How much do you agree with the following statements about the **COVID-19 virus in children**?
       1. *Statements:*
          1. I am worried about the possibility of my [child]/[children] getting COVID-19.
          2. Attending daycare or school in person could increase my [child’s]/[children’s] risk of getting COVID-19.
          3. It would be bad if my [child]/[children] got COVID-19.
          4. If my [child]/[children] got COVID-19, [he/she]/[they] could get very sick.
          5. [My child]/[One or more of my children] is at greater risk for getting very sick from COVID-19 than the average child.
          6. Children could spread COVID-19 to someone else and make them very sick.
          7. I am worried about how new COVID-19 variants affect children.
          8. I am worried that I could give my [child]/[children] COVID-19.
       2. *Responses:*
          1. Very strongly agree
          2. Strongly agree
          3. Somewhat agree
          4. Do not agree
   13. How much do you agree with the following statements about the **COVID-19 virus for yourself**?
       1. *Statements:*
          1. I am worried about the possibility of getting COVID-19 myself.
          2. Attending work or school in person could increase my risk of getting COVID-19.
          3. It would be bad if I got COVID-19.
          4. If I got COVID-19, I could get very sick.
          5. I am at greater risk for getting very sick from COVID-19 than the average adult.
          6. I could spread COVID-19 to someone else and make them very sick.
          7. I am worried about how new variants affect adults.
          8. I am worried that my [child]/[children] could give me COVID-19.
       2. *Responses:*
          1. Very strongly agree
          2. Strongly agree
          3. Somewhat agree
          4. Do not agree
4. [*If parent of child ages 12-17 🡪*] The rest of this survey will focus on vaccines against COVID-19.
   1. [*If parent of child ages 12-17 🡪*] [Has **your child**]/[Have any of **your children**] **aged 12 to 17** received **at least one dose** of a COVID-19 vaccine?
      - 1. Yes
        2. No
   2. [*If answer to Q16 was “No” or refused 🡪*] How likely are you to get **your** [**child**]/[**children**] **aged 12 to 17** vaccinated against COVID-19?
      - 1. Very likely
        2. Somewhat likely
        3. Somewhat unlikely
        4. Very unlikely
5. [*If parent of child ages 5-11 🡪*] The next questions will ask about your opinions of COVID-19 vaccines for **children aged 5 to 11.**
   1. [*If parent of child ages 5-11 🡪*] [Has **your child**]/[Have any of **your children**] **aged 5 to 11** received **at least one dose** of a COVID-19 vaccine?
      - 1. Yes
        2. No
   2. [*If answer to Q18 was “No” or refused 🡪*] How likely are you to get **your** [**child**]/[**children**] **aged 5 to 11** vaccinated against COVID-19?
      - 1. Very likely
        2. Somewhat likely
        3. Somewhat unlikely
        4. Very unlikely
   3. [*If answer to Q18 was “No” or refused 🡪*] If a COVID-19 vaccine is authorized for use in children between 5 and 11 years old, when would you be willing to get **your** [**child**]/[**children**] **aged 5 to 11** vaccinated against COVID-19?
      - 1. As soon as possible once the vaccine is authorized for and available for children
        2. After the vaccine is authorized for children and a **small** number of children have gotten vaccinated
        3. After the vaccine is authorized for children and a **large** number of children have gotten vaccinated
        4. I would not get my [child]/[children] vaccinated against COVID-19
6. [*If parent of child ages 0-4 🡪*] The next questions will ask about your opinions of COVID-19 vaccines for **children aged 0 to 4.**
   1. [*If parent of child ages 0-4 🡪*] If a safe, effective COVID-19 vaccine were available for children 4 and younger, how likely would you be to get **your** [**child**]/[**children**] **aged 0 to 4** vaccinated against COVID-19?
      - 1. [My child]/[At least one of my children] already got vaccinated as part of a trial
        2. Very likely
        3. Somewhat likely
        4. Somewhat unlikely
        5. Very unlikely
   2. [*If answer to Q21 was “Very likely,” “Somewhat likely,” “Somewhat unlikely,” “Very unlikely,” or refused 🡪*] If a COVID-19 vaccine is authorized for use in children 4 and younger, when would you be willing to get **your** [**child**]/[**children**] **aged 0 to 4** vaccinated against COVID-19?
      - 1. As soon as possible once the vaccine is authorized for and available for children
        2. After the vaccine is authorized for children and a **small** number of children have gotten vaccinated
        3. After the vaccine is authorized for children and a **large** number of children have gotten vaccinated
        4. I would not get my [child]/[children] vaccinated against COVID-19
7. The next questions will ask about your opinions of COVID-19 vaccines for **children in general**.
   1. How much do you agree with the following statements about COVID-19 vaccines?
      1. *Statements:*
         1. COVID-19 vaccines have been developed too quickly.
         2. I am concerned about possible serious side effects of COVID-19 vaccines in children.
         3. Being able to give my [child]/[children] a COVID-19 vaccine would make me more comfortable sending them to daycare or school.
         4. My [child’s]/[children’s] healthcare provider is a reliable and trustworthy source of information about COVID-19 vaccines.
         5. The COVID-19 vaccine may not work to prevent COVID-19 in children.
         6. Getting a COVID-19 vaccine would protect my [child]/[children] from COVID-19.
         7. Having my [child]/[children] vaccinated against COVID-19 would protect the health of others in my community.
         8. I am concerned about possible rare side effects of COVID-19 vaccines in children.
         9. Most children do not need a COVID-19 vaccine.
      2. *Responses:*
         1. Very strongly agree
         2. Strongly agree
         3. Somewhat agree
         4. Do not agree
   2. [*If parent of unvaccinated child 🡪*] Would the following make you more or less likely to have your unvaccinated [child]/[children] get a COVID-19 vaccine?
      1. *Statements:*
         1. If my [child’s]/[children’s] healthcare provider recommends it
         2. If I know a lot of people of all ages who have gotten it
         3. If it is required to return to school or daycare
         4. If it is required to travel
         5. If it is given at the same time as a routine vaccine
         6. If it causes the same or fewer short-term side effects than routine vaccines
         7. If it causes more severe side effects, such as high fever or more arm pain, than routine vaccines
         8. If I know a lot of children who have gotten it
         9. If a different type of vaccine becomes available for children
         10. If a vaccine receives **full FDA approval** rather than just emergency use authorization
         11. If I get paid time off work
         12. If I get free childcare assistance
         13. If I get free transportation
         14. If I am encouraged by local religious or community leaders
      2. *Responses:*
         1. Much more likely
         2. Somewhat more likely
         3. No more or less likely
         4. Somewhat less likely
         5. Much less likely
   3. [*If parent of child ages 12-17 🡪*] [When you were]/[As you are] thinking about if you should get [**your child**]/[any of **your children**] **aged 12 to 17** vaccinated against COVID-19, how much influence [did]/[do] the following have on your decision?
      1. *Statements:*
         1. Desire to protect my child from COVID-19
         2. [*If answer to Q11 was “Yes” 🡪*] Desire to protect my child from MIS-C
         3. Concern about my child feeling sick after the vaccine
         4. Concern about my child having serious vaccine side effects
         5. Distrust of the healthcare system
         6. Religious concerns about the vaccine
         7. Desire to protect others from getting infected from my child
         8. Wanting my child to be able to attend school or camp
         9. Wanting my child to be able to travel freely
         10. My child’s opinion about getting it
         11. My child’s healthcare provider’s recommendation about getting it
         12. My friends’ decisions about getting their children vaccinated
         13. The kinds of vaccines available
         14. Desire to protect my child against new COVID-19 variants
      2. *Responses:*
         1. A lot of influence
         2. Some influence
         3. No influence
   4. [*If parent of child ages 5-11 🡪*] [When you were]/[As you are] thinking about if you should get [**your child**]/[any of **your children**] **aged 5 to 11** vaccinated against COVID-19, how much influence [did]/[do] the following have on your decision?
      1. *Statements:*
         1. Desire to protect my child from COVID-19
         2. [*If answer to Q11 was “Yes” 🡪*] Desire to protect my child from MIS-C
         3. Concern about my child feeling sick after the vaccine
         4. Concern about my child having serious vaccine side effects
         5. Distrust of the healthcare system
         6. Religious concerns about the vaccine
         7. Desire to protect others from getting infected from my child
         8. Wanting my child to be able to attend school or camp
         9. Wanting my child to be able to travel freely
         10. My child’s opinion about getting it
         11. My child’s healthcare provider’s recommendation about getting it
         12. My friends’ decisions about getting their children vaccinated
         13. The kinds of vaccines available
         14. Desire to protect my child against new COVID-19 variants
      2. *Responses:*
         1. A lot of influence
         2. Some influence
         3. No influence
   5. [*If parent of child ages 0-4 🡪*] [When you were]/[As you are] thinking about if you should get [**your child**]/[any of **your children**] **aged 0 to 4** vaccinated against COVID-19, how much influence [did]/[do] the following have on your decision?
      1. *Statements:*
         1. Desire to protect my child from COVID-19
         2. [*If answer to Q11 was “Yes” 🡪*] Desire to protect my child from MIS-C
         3. Concern about my child feeling sick after the vaccine
         4. Concern about my child having serious vaccine side effects
         5. Distrust of the healthcare system
         6. Religious concerns about the vaccine
         7. Desire to protect others from getting infected from my child
         8. Wanting my child to be able to attend school or daycare
         9. Wanting my child to be able to travel freely
         10. My child’s opinion about getting it
         11. My child’s healthcare provider’s recommendation about getting it
         12. My friends’ decisions about getting their children vaccinated
         13. The kinds of vaccines available
         14. Desire to protect my child against new COVID-19 variants
      2. *Responses:*
         1. A lot of influence
         2. Some influence
         3. No influence

The next few questions will ask about your opinions of COVID-19 vaccines **for yourself**.

- 1. Have you received **at least one dose** of a COVID-19 vaccine?
     - 1. Yes
       2. No
  2. [*If answer to Q28 was “No” or refused 🡪*] How likely are you to get **yourself** vaccinated against COVID-19?
     - 1. Very likely
       2. Somewhat likely
       3. Somewhat unlikely
       4. Very unlikely
  3. [When you were]/[As you are] thinking about if **you** should get vaccinated against COVID-19, how much influence [did]/[do] the following have on your decision?
     1. *Statements:*
        1. Desire to protect myself from COVID-19
        2. Concern about feeling sick after the vaccine
        3. Concern about having serious vaccine side effects
        4. Distrust of the healthcare system
        5. Religious concerns about the vaccine
        6. Desire to protect others from getting infected from me
        7. Wanting to be able to go to work or school
        8. Wanting to be able to travel freely
        9. My healthcare provider’s recommendation about getting it
        10. My friends’ decisions about getting vaccinated
        11. The kinds of vaccines available
        12. Desire to protect myself against new COVID-19 variants
        13. Rewards or entry into a lottery
     2. *Responses:*
        1. A lot of influence
        2. Some influence
        3. No influence
  4. [*If answer to Q28 was “No” or refused 🡪*] Would the following make you more or less likely to get a COVID-19 vaccine **for yourself**?
     1. *Statements:*
        1. If my healthcare provider recommends it
        2. If I know a lot of people who have gotten it
        3. If it is required to return to work or school
        4. If it is required to travel
        5. If it is given at the same time as a routine vaccine
        6. If it causes the same or fewer short-term side effects than routine vaccines
        7. If it causes more severe side effects, such as high fever or more arm pain, than routine vaccines
        8. If a different type of vaccine becomes available
        9. If a vaccine receives full FDA approval rather than just emergency use authorization
        10. If I am encouraged by local religious or community leaders
        11. If I get paid time off work
        12. If I get free childcare assistance
        13. If I get free transportation
     2. *Responses:*
        1. Much more likely
        2. Somewhat more likely
        3. No more or less likely
        4. Somewhat less likely
        5. Much less likely

1. Next, we'd like to ask some questions about you and your [child]/[children].
   1. How old are you?

      *Please confirm your age. Type in your age if the box is empty or the age shown is incorrect.*
      - 1. [TEXTBOX] years old
   2. Are you…?

      *Please change if response shown below is incorrect.*
      - 1. Male
        2. Female
   3. Does [your child]/[one or more of your children] have a chronic health condition?
      - 1. Yes
        2. No
   4. [*If answer to D3 was “Yes” 🡪*] Do you believe that this chronic health condition could put [your child]/[one or more of your children] at a higher risk for COVID-19?
      - 1. Yes
        2. No
   5. Do **you** have a chronic health condition?
      - 1. Yes
        2. No
   6. [*If answer to D5 was “Yes” 🡪*] Do you believe that this chronic health condition could put **you** at a higher risk for COVID-19?
      - 1. Yes
        2. No
   7. Are you a healthcare worker?
      - 1. Yes
        2. No
   8. [*If answer to D7 was “Yes” 🡪*] Which one of the following best describes you?
      - 1. Nurse
        2. Advanced Practice Provider (NP, PA, etc.)
        3. Physician
        4. Another kind of healthcare worker
   9. Do you have any comments about this survey?
      - 1. [TEXTBOX]
        2. No comment
2. Please click the next button to complete the survey.
